# Genomic and metagenomic signatures of giant viruses are ubiquitous in water samples from sewage, inland lake, waste water treatment plant, and municipal water supply in Mumbai, India

**DOI:** 10.1038/s41598-019-40171-y

**Published:** 2019-03-06

**Authors:** Anirvan Chatterjee, Thomas Sicheritz-Pontén, Rajesh Yadav, Kiran Kondabagil

**Affiliations:** 1 Department of Biosciences and Bioengineering, Indian Institute of Technology Bombay, Powai, Mumbai, India; 2 Centre of Excellence for Omics-Driven Computational Biodiscovery (COMBio), Faculty of Applied Sciences, AIMST University, Kedah, Malaysia

## Abstract

We report the detection of genomic signatures of giant viruses (GVs) in the metagenomes of three environment samples from Mumbai, India, namely, a pre-filter of a household water purifier, a sludge sample from wastewater treatment plant (WWTP), and a drying bed sample of the same WWTP. The *de novo* assembled contigs of each sample yielded 700 to 2000 maximum unique matches with the GV genomic database. In all three samples, the maximum number of reads aligned to Pandoraviridae, followed by Phycodnaviridae, Mimiviridae, Iridoviridae, and other Megaviruses. We also isolated GVs from every environmental sample (n = 20) we tested using co-culture of the sample with *Acanthomoeba castellanii*. From this, four randomly selected GVs were subjected to the genomic characterization that showed remarkable cladistic homology with the three GV families viz., Mimivirirdae (*Mimivirus Bombay [MVB])*, Megaviruses (*Powai lake megavirus [PLMV]* and *Bandra megavius [BAV])*, and Marseilleviridae (*Kurlavirus [KV])*. All 4 isolates exhibited remarkable genomic identity with respective GV families. Functionally, the genomes were indistinguishable from other previously reported GVs, encoding nearly all COGs across extant family members. Further, the uncanny genomic homogeneity exhibited by individual GV families across distant geographies indicate their yet to be ascertained ecological significance.

## Introduction

The discovery of *Acanthamoeba polyphaga mimivirus* (APMV)^1,2^ galvanized the search for other giant viruses (GVs). Subsequently, GVs have been isolated from diverse environmental niches, including cooling towers, sewage, fresh water, and coastal water^3^. In fact, nucleocytoplasmic large DNA viruses (NCLDVs) in the photic layer of oceans were thought to outnumber the eukaryotic organisms^4^. Metagenomic identification of *Klosneuvirus*, a new GV family, from wastewater treatment plant (WWTP) and their detection in the existing environmental metagenomes indicated their previously undetected presence^5^. Despite the discovery of several GV families, very little is known about their natural hosts, their role in the ecology, and biogeochemical pathways. While the Phycodnaviridae members are believed to control the planktonic communities^6^, the role of other GVs in their environment is largely unknown.

The current classification of NCLDVs consists of six closely related families of amoebal megaviruses, namely, Mimiviridae, Marseilleviridae, Pandoraviridae, Pithoviridae, Faustoviridae, and Molliviridae^3^. While the evolutionary genealogy of NCLDVs remains highly debated^7–11^, the comparative genomics of several new amoebal NCDLV genomes from diverse geographies have augmented their accurate familial classification^12–17^. Both genome *expansion*^18,19^ and *reduction*^20^ models have been explored for explaining the evolution of the large genomes of NCLDVs. Diverse genetic processes such as horizontal and lateral gene transfers, multiple episodes of gene loss/gain, duplication, transposition, and insertion have been observed across NCLDV genomes^21^. Sequencing of more NCLDV genomes helped in recognizing the true complexity of NCLDVs, which in addition to asserting the presence of a common ancestor with a smaller set of genes, revealed the immense variability^22^. The amino-acyl tRNA synthetases have been found to be duplicated in *Niemeyer virus*^15^ but absent in Faustoviruses^23^. Further, less than a quarter of the faustoviral genes matched with other NCLDVs while ~46% were homologous to bacterial genes and the remaining genes were ORFans^13^ exhibiting greater diversity. The phylogenetic analysis of some NCLDV core proteins such as the primase-helicase fusion proteins indicated their complex evolutionary histories^24^, while the DNA packaging machinery was thought to be of bacterial origin^25,26^. Furthermore, Mollivirus, a distinct member of the NCLDV family, was found to lack the crucial DNA biosynthesis enzyme, ribonucleotide reductase, that is ubiquitously found in other amoebal NCLDVs^14^. The lineage-specific genealogies have also been shown to be critical for understanding the evolution of these viruses. For example, the number of genes encoding Repeat Domain-Containing Proteins (RDCPs) in the genomes of amoebal viruses are thought to be one of the major drivers of genome evolution and its plasticity^27^. Thus, finding new NCLDVs and their genomic signatures in diverse environments would help in understanding their diversity, abundance, and ecological significance.

Here, we report the detection of NCLDV genomic signatures in the metagenomes from a municipal household water supply (a pre-filter from a water purifier), and, sludge and drying bed samples from a wastewater treatment plant (WWTP) of a dairy. In addition, we describe the genomic features of four new amoebal viruses isolated from sewage, urban water drain, and an inland lake in Mumbai. These viruses exhibit significant genome rearrangements when compared to other GVs, yet they maintain functional conservation, indicating a purifying selection by their host and environment. While we expected the ubiquitous presence of GVs in the samples and their genomic signatures in random metagenomes, the discovery of three different GV lineages, with remarkable functional conservation with GVs isolated from distant continents warrants the need for understanding their role in the ecology.

## Results

**Figure 1:**
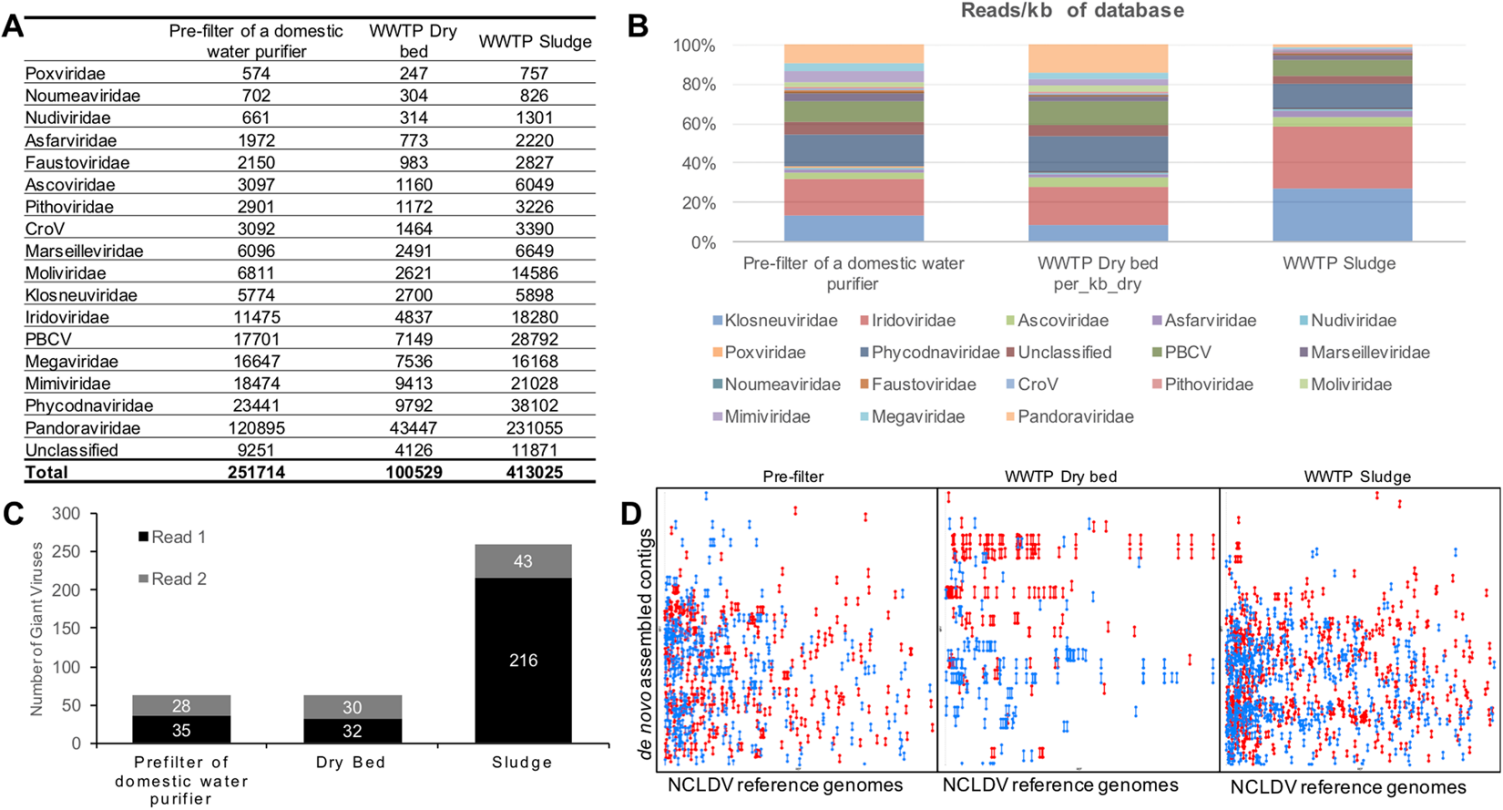
Genomic signatures of NCLDVs in the 3 metagenomes. (**A**) Total number of reads mapped to each NCLDV family. (**B**) Proportion of normalised read count assigned to each NCLDV family. (**C**) Number of NCLDVs detected in each metagenome as determined by Giant Virus Finder^61^. For each metagenome, GVF was run independently for both reads (from Illumina paired-end sequencing). (**D**) Maximum unique matches between the *de novo* assembled contigs of each metagenome (plotted on Y-axis) with NCLDV genomes. Each red line indicates a forward match of at least 200 nucleotides, a reverse match of at least 200 nucleotides is represented by a blue line. The contigs represented on the Y-axes were assembled from NCLDV reads selected in (A) pre-filter of a domestic water purifier (B) Dairy WWTP drying bed, and (C) Dairy WWTP sludge.

The rapidly expanding database of NCLDV genomes has enabled detection of their genomic signatures in the diverse metagenomic datasets^5^. We performed metagenomics of two samples from WWTP of a dairy and one sample from the pre-filter of a domestic water purifier. As described in the *Methods* section, MGmapper was used to identify reads matching to bacteria, archaea, and protozoa. As expected, bacterial reads were found to be the most abundant in all samples. We also detected the genomic signatures of several protozoa, including *Acanthamoeba spp*. in all 3 metagenomes (Table 1). Further, about 7% of the reads from all samples aligned to the in-house NCLDV genome database (see *Methods*). Samples from pre-filter, WWTP sludge, and WWTP drying bed contained 251714 (6.7%), 100529 (6.4%), and 413025 (7.7%) reads which aligned to NCLDVs, respectively (Fig. 1A). Read-counts normalised for genomic database size indicated maximum relative abundance of reads mapping to *Kloseneuviridae* and *Iridoviridae* (Fig. 1B). Further, stringent (*e-value* < *1e-4, word size* = 7) search using BLAST-based GVF was performed on both reads (from Illumina paired-end sequencing) against the GV database (Supplementary Table 1). Cumulatively, we detected the presence of 63, 62, and 259 distinct GVs in the pre-filter, WWTP drying bed, and WWTP sludge samples, respectively (Fig. 1C). The consolidated list of Blast hits against giant viruses is provided in the Supplementary Tables 2–4. As described in previous studies^4,5^, we observed that the reads aligning to NCLDVs exhibited low-complex nucleotide content. Further, we explored maximum unique matches (MUMs) between the *de novo* assembled contigs of each sample to the NCLDV database and observed that the number of MUMs ranged between 700 to 2000 (Fig. 1D). In congruence with the results from read-alignments and GVF, the nucleotide matches with the *de novo* assemblies showed maximum matches with sludge from WWTP.

**Table 1:** Number of reads mapping to Bacteria, Archaea and Protozoa in metagenomes from filter of house water purifier and, dry-bed and sludge of waste water treatment plant of a dairy in Mumbai.

	House filter	WWTP Dry bed	WWTP Sludge
Bacteria	83326	45774	1263646
Archaea	142	2676	2906
Protozoa	11110	5924	168780

The mapping was MGmapper (Petersen *et al*. ^52^) in the *‘Full Mode’ (-F 1,2,10*).

**Figure 2:**
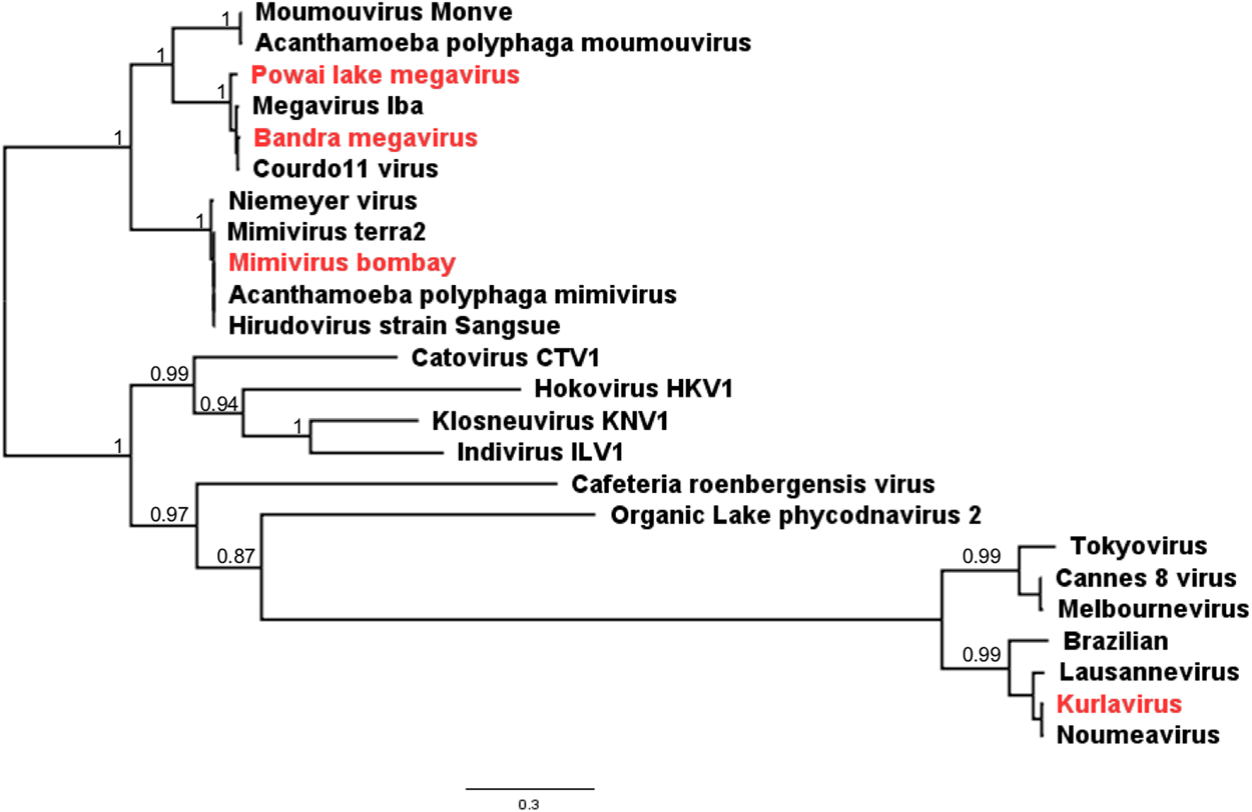
Maximum likelihood phylogeny based on DNA pol B, classifying genomes of the 4 new NCLDVs isolates discovered in the environmental samples from Mumbai, India, into 3 families.

We also isolated several GVs using *A. castellanii* as host from several environmental water samples around Mumbai. Purified viral particles exhibited icosahedral morphology and size ranged from 150 nm to 480 nm. To further characterize the viruses, we performed whole-genome sequencing of 4 viral isolates from 4 different samples, including the smallest isolate (150 nm) and three particles >400 nm in diameter. We reported the genome sequence of three viruses earlier, namely, *Powai lake megavirus* (PLMV)^28^, *Mimivirus bombay* (MVB)^29^, and *Kurlavirus* (KUV)^30^. Here, we report the genome sequence of *Bandra megavirus* (BMV), the fourth NCLDV reported from India, which was found to be phylogenetically related to the other Megaviruses (Fig. 2). BMV particles were found to be about 465 nm in diameter, the largest of the GVs reported by us.

**Figure 3:**
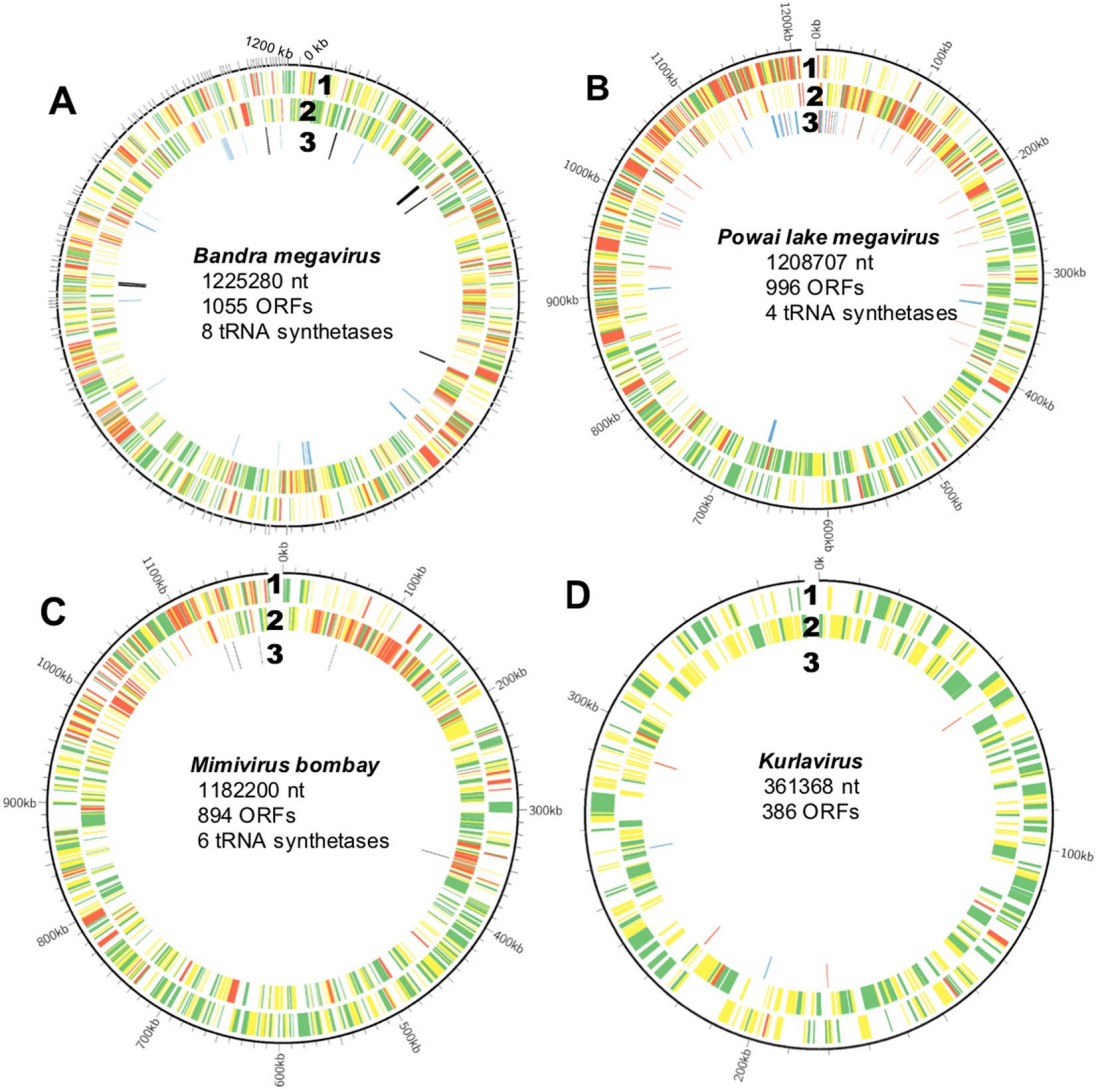
Circos ideogram depicting genome characteristics of (**A**) *Powai lake megavirus*, (**B**) *Bandra megavirus*, (**C**) *Mimivirus Bombay* and (**D**). *Kurlavirus*. The linear genomes are represented as the outermost concentric circular line. From the outermost, the first and second concentric circle indicate genes on leading and lagging strand respectively. In 1 and 2 the colors denote: Red: Genes encoding Repeat Domain Containing Proteins; Yellow: Genes annotated as hypothetical protein; Green: Genes with known/putative functions. The third concentric (3) consist of: Black: Genes encoding tRNA sythetases; Red: Genes with maximum alignment score with other bacterial homologues; Orange: Genes with maximum alignment score with eukaryotic homologues; Blue: Genes with maximum alignment score with homologues in NCLDVs other than its own family.

With a length of 1,235,891 bp, the draft genome of BMV is the largest GV genome reported from India as compared to other Indian isolates (Fig. 3). Consequently, BAMV had the maximum number of predicted ORFs (n = 1055) with 544 ORFs on the leading strand and 511 ORFs on the lagging strand (Fig. 3A). We classified the predicted ORFs into 3 broad annotated groups, viz. known or putative function, hypothetical proteins, and repeat domain-containing proteins (RDCPs). RDCPs were classified independent of other functional classes because of their succinct role in protein evolution^27,31,32^ and their conspicuous presence in the genomic termini of GVs^33^. The KUV genome had the least number of RDCPs (n = 6), whereas >15% of the ORFs in the other three GVs encoded RDCPs. Like other *Marseilleviridae*, KUV had a high GC content of 42.9%, as compared to the low GC scores of 25.3%, 27.9% and 25.2% in PLMV, MVB, and BMV, respectively. While KUV encoded no tRNA genes as expected, BMV was found to encode most number of tRNA synthetases (n = 8) followed by 6 in MVB and 5 in PLMV. Further, KUV was found to encode only one capsid protein, as compared to 4 by other 3 GVs. Typical of other *Marseilleviridae*¸ the KUV genome encoded 3 histone-like proteins. A phylogeny based on the concatenated histone-like proteins placed KUV in *Marseilleviridae* Lineage B^34^, closely related to *Noumeavirus* (Supplementary Fig. 1).

**Figure 4:**
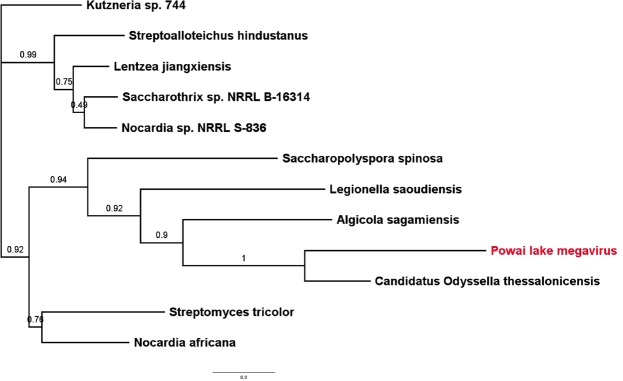
Maximum likelihood phylogeny of tRNA-dependent cyclodipeptide synthase gene in PLMV indicating homology with gene previously reported in *Candidatus Odyssella thessalonicensis*, an endosymbiont infecting the *Acanthamoeba spp*.

A large number of genes in GVs are predicted to be related to genes of the diverse group of cellular organisms leading to speculations of their association with diverse hosts in the environment^4,35^. Many such genes have been found to be conserved across GVs, and are now classified as NCVOGs^9,36^. Both PLMV and BMV encoded ORFs that showed maximum identity with homologs in bacteria and eukarya (Fig. 3; Innermost concentric circle). In KUV, 2 ORFs showed maximum identity with eukaryotic homologs and 1 ORF showed maximum alignment score [e-value = 6e-62] with a bacterial homolog. The PLMV genome had 30 ORFs with a maximum alignment score with other bacterial homologs compared to homologs in other megaviruses. Of the 2 ORFs in PLMV which showed maximum alignment with eukaryotic homologs, 1 ORF encoded a hypothetical protein and the other encoded a tRNA-dependent cyclodipeptide synthase, an enzyme not reported so far from any virus. A phylogenetic analysis of the gene (Fig. 4) revealed it to be closely related to its homologue in *Candidatus Odyssella thessalonicensis*, an endosymbiont infecting the *Acanthamoeba* spp.^37^.

**Figure 5:**
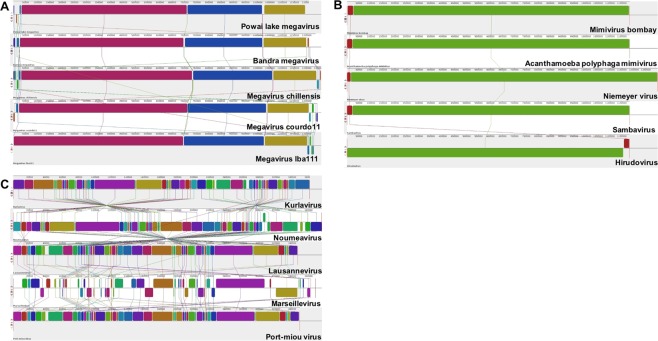
Whole genome alignment linear collinear blocks (LCBs), showing synteny across isolates belonging to the same lineage. (**A**) LCBs in Powai lake megavirus and *Bandra megavirus* with 3 other Megaviruses, *viz. Megavirus chiliensis*, *Megavirus courdo11* and *Megavirus lba111*. (**B**) LCBs in Mimivirus bombay and 4 other Mimiviridae, *viz. Acanthamoeba polyphaga mimivirus*, *Niemeyer virus*, *Sambavirus* and *Hirudovirus*. (**C**) LCBs in Kurlavirus and 4 other Marseilleviridae, *viz. Noumeavirus*, *Lausannevirus*, *Marseillevirus* and *Port-miou virus*.

All genomes showed remarkable genomic similarity with other members of the same lineage isolated from distant geographies. Thus, we compared the genomes of each of the viruses with 4 other genomes from the closest phylogenetically related GVs based on the B family DNA polymerase (Fig. 5A–C). Whole genome alignments based on Mauve^38^ were used to identify *locally collinear blocks* (LCBs), which showed maximal homology among the genomes but with internal rearrangements. The PLMV genome was aligned with three other megaviruses, viz., *M.chiliensis*, *M.lba111*, and *M.courdo11* showed greater synteny towards the centre of the linear genome (Fig. 5A). The genomic termini exhibited several rearrangements, while the LCBs were largely common in all genomes. We observed similar synteny when the genome of BAMV was compared to PLMV, *M.chilensis*, *M.lba111*, and *M.courdo11* (Fig. 5A). MVB showed maximum genomic synteny wherein the genomes of MVB, *Acanthamoeba mimivirus*, *Sambavirus*, *Niemeyer virus*, and *Hirudovirus* aligned into just 2 LCBs (Fig. 5B). Interestingly, maximum genomic variation was observed when we aligned the whole genomes of KUV, *Noumeavirus*, *Lausannevirus*, *Marseillevirus*, and *Port-miou* virus. In fact, *Marseillevirus*, the founding member of the *Marseilleviridae* family, showed least genomic homology with other members of the *Marseilleviridae* family (Fig. 5C). As seen in the phylogeny (Fig. 3), KUV showed maximum genomic homology with *Noumeavirus*. Unlike the other 3 viruses, we could not identify any synteny in KUV and other Marseilleviridae family members. The variability among the members of the same lineage was further observed in plots depicting the maximal unique nucleotide matches (*nucmer*)^39^. We observed several genomic gaps in all alignments (Supplementary Fig. 2A–D).

## Discussion

Straddled between cellular life forms and simpler viruses, GVs and their ecological niche is a theme of intense research^8^. The discovery of GVs from diverse geographies is critical for deciphering their evolutionary history. Recent studies have used culture free systems for detecting NCLDV genomic signatures in the metagenomes of diverse environments^40–42^. Here, we report the presence of NCLDV genomic signatures in metagenomes extracted from a pre-filter of a domestic water purifier and WWTP. We demonstrated the ubiquitous presence of GVs in diverse environmental samples, including drinking water supply in an urban metropolis such as Mumbai. Pandoraviridae yielded maximum read matches, and the normalised read counts indicated maximum read matches to *Klosneuviridae*, which was first isolated from sewage samples^5^. This augments the diversity of GVs in environmental samples in the region, wherein a co-culture with *A. castellanni* GVs closely related to mimivirus, megavirus and marseilleviridae and, culture free approaches revealed the presence of several viral species for with no known laboratory hosts. Being part of the metagenomic *dark matter*, these viruses may only be detected by culture independent methods. Despite detecting several genomic hits to the exhaustively curated NCLDVs database, full length NCLDV genes could not be assembled. In future studies, a size based fractionation of the sample may enable independent measure of bacterial, viral and protozoan diversity.

We isolated several GVs using amoebal co-culture. The sequenced GV isolates of Mumbai belonged to the 3 of the most abundant GV families, viz. *Mimiviridae*, *Megaviridae*, and *Marseilleviridae*. While the phylogenetic reconstruction of the 4 viruses was unambiguous in their cladistic placement (Fig. 2), there were large-scale genome rearrangements, indicating high plasticity (Fig. 5). The 4 novel GVs reported here, exhibited extraordinary congruence with hallmark features of their respective GV families. Exclusive occurrence of genes encoding histone-like proteins and the absence of tRNAs in KV, a *Marseilleviridae*, substantiates the proposed monophyletic origin of GVs. The functional conservation of GVs across different geographies indicates a significant role in the microbial ecology which is yet to be ascertained. Despite more than a decade of research on GVs, natural hosts of many GV families are yet to be established. While co-culturing with *Acanthamoeba spp*. has augmented isolation of GVs, much of the NCLDVs which do not infect *Acanthamoeba spp*. remains unstudied.

The extreme genetic mosaicism seen in these viruses indicate that their life cycle gives them access to genes from all three branches of life, making them a source and recipient of genetic exchange in the environment. In PLMV, ORF 45 is annotated as tRNA-dependent cyclodipeptide synthase based on sequence identity with a homolog found in the *Candidatus Odyssella thessalonicensis*, an endosymbiont of *Acanthamoeba* spp.^37^. This is the first tRNA-dependent cyclodipeptide synthase to be reported from an NCLDV. The tRNA-dependent cyclodipeptide synthase is thought to be a paralog of aminoacyl–tRNA synthetases^43^ which catalyzes the synthesis of cyclopeptides^44^. This extends the genomic repertoire of *Mimiviridae* beyond the translational genes reported in Tupanvirus^45^. A near complete sequence identity with *Candidatus Odyssella thessalonicensis* cyclopeptide synthase and its unique presence in PLMV suggests a lateral acquisition in their common host (*Acanthamoeba spp.)*. Several such gene families have been shown to be laterally acquired from diverse species including viruses, bacteria, archaea and eukarya, resulting from an apparent mobilome^24,46,47^. By way of facilitating genetic exchange and/or controlling the population of their hosts, GVs could be crucial in the microbial ecology. While the currently available data are insufficient to choose between a genomic accretion and reduction model^45^, the extreme functional conservation within the each GV family across distant geographies, despite large-scale genomic rearrangements, indicates a niche/host-specific adaptation.

Giant viruses have been primarily studied to ascertain their true classification^8^ and evolutionary significance^9,48,49^. More recently, GVs have been detected in humans, associated with respiratory illness^50^, cancer^51^. In addition to isolation of GVs, metagenomic studies have contributed significantly to our understanding of NCLDV diversity and abundance, and also their detection in environments that are shared with human communities^5^. Results presented here and in other recent reports^40–42^ assert their previously undetected ubiquity in diverse environments. Exploring functional networks of NCLDVs in viromes and their co-occurrence with other species is essential to understand their fundamental role in microbial ecology.

## Materials and Methods

### Metagenomics

#### Sample processing and DNA extraction

Samples were collected from the solid deposits on a pre-filter of a commercially available domestic water purifier (referred to as pre-filter); and drying bed and sludge of the wastewater treatment plant (WWTP) of a dairy industry in Mumbai. The pre-filter was used for 3 months and the deposits are from about 2000 L of water. Dry samples (0.25 g) were processed using Power Soil DNA extraction kit (Mobio, USA) as per the manufacturer’s instructions. Fifteen-ml sludge sample was treated with 10% polyethylene glycol (PEG) 6000 overnight at 4 °C followed by centrifugation at 5000 *g* for 60 min and the virus-enriched precipitate was used for DNA extraction using the Power Soil DNA extraction kit (Mobio, USA). The total amount of DNA extracted was between 20 and 80 ng. DNA was further concentrated using Vacufuge (Eppendorf, Germany), and re-suspended in 10 µl DEPC treated water.

#### Whole genome shotgun sequencing

Whole genome shotgun sequencing was performed using Miseq (Illumina Inc, USA) as per the manufacturer’s instructions. Five-µl of the extracted DNA (concentration 0.2 ng/µl) was used for library preparation (fragmentation and tagmentation) using Nextera XT (Illumina Inc. USA). Normalized libraries were sequenced using 2 × 150 Miseq V2 kit. Raw data were processed using Basecaller to generate paired fastq files.

#### Metagenomic read binning

Primary metagenomics analysis was performed using *MGmapper*^52^. Reads from all samples were assigned to 3 databases (bacteria, archaea, and protozoa) in the *‘Full Mode’ (-F 1,2,10)* with other default parameters. Post read-filtering (QV > 30), adapter trimming, and de-duplication, quality of the data was ascertained using FastQC (https://www.bioinformatics.babraham.ac.uk/projects/fastqc/). *MGmapper* could not be used to identify reads belonging to giant viral families since these reads have been shown to be non-complex in nature^5^, exhibiting multiple stretches of di-, tri- nucleotide repeats. To extract reads aligning with NCLDVs, a database was generated by manually curating all genome sequences downloaded from NCBI database classified as *Poxviridae*, *Noumeaviridae, Nudiviridae, Asfarviridae, Faustoviridae, Ascoviridae, Pithoviridae, Marseilleviridae, Moliviridae Klosneuviridae, Unclassified Iridoviridae, Phycodnaviridae, Megaviridae, Mimiviridae*, and *Pandoraviridae*. Reads from each sample were aligned with this custom NCLDV database using three aligners, viz., Bowtie^53^, BBMap (https://sourceforge.net/projects/bbmap/), and BWA-MEM^54^, and filtered using Samtools^55^. To determine the best aligner, the extracted reads were subjected to *de novo* assembly using metaSpades^56^, MetaVelvet^57^ and IDBA-UD^58^, and evaluated using QUAST^59^, a tool for quality assessment of genome assemblies for previously unsequenced species. We observed that the NCLDV reads extracted using BWA-MEM yielded contigs with the best N50, which were further used to find maximum unique matches with the NCLDV database. While current genomic databases limit quantification of viral abundance from shotgun metagenomes, normalised read-counts are employed for taxonomic classification^60^. Read-counts across the three samples were normalised based on their relative abundance per 1 kb of genomic database^60^. In absence of a conserved indicator gene, we used the *reads per kilobase per genome* (RPKG) strategy to normalise the data^60^.

We used the Giant Virus Finder (GVF) pipeline^61^ as a secondary analysis tool to confirm the presence of NCLDV genomic signatures. The pipeline was locally setup and performed as per the instructions. A database of non-redundant (NR) and nucleotide (NT) of all NCLDV genomes was locally setup. Using the GVF, a blast database of viruses with genome sizes greater than 300,000 bp (List of viruses in Supplementary Table 1) was generated and used to extract the reads with an e-value < *1e-4*. Extracted reads were remapped to the NT database with an e-value < *1e-4* and the hits were enumerated (Supplementary Tables 2–4).

### Virus purification and genome extraction

In addition to the metagenomic analysis of the 3 samples, several other samples were analysed to detect, isolate, and characterize giant viruses in the environmental samples in Mumbai. These samples were collected independent of the samples used for metagenomic analysis. Thus, classical microbiology was used to enrich giant viruses in samples followed by co-culture in *Acantamoeba castellanii* and purification of viral lysates using a sucrose gradient as described earlier^30^.

The purified viral fraction obtained from sucrose density gradient was used for DNA extraction by the phenol-chloroform method, followed by ethanol precipitation^1^. Briefly, virus particles were disrupted by heating at 90 °C followed by enzymatic digestion using lysozyme. Proteins were digested using Proteinase K and SDS and separated using two repetitions of phenol-chloroform separation. Phenol was removed using chloroform-isoamyl alcohol (24:1). DNA was purified using ethanol-salt precipitation. DNA quality and quantity were ascertained by spectrophotometric and electrophoretic methods.

#### Whole genome shotgun sequencing, genome assembly, annotation and analysis

WGS was performed as reported earlier^28–30^. Raw reads were filtered for QV > 30. SureSelect^QXT^ tags were trimmed using SureCall suite (Agilent Technologies). FastQC of pre- and post-trimming and filtering were compared. *De novo* assembly was performed using multiple assemblers including SOAPdenovo2^62^, A5-miseq^63^, and Velvet^57^, and evaluated using QUAST^59^. MAUVE^38^ was used to reorder the contigs and generate consensus FASTA. Open reading frames (ORFs) were predicted with GeneMarkS^64^, individually annotated using Blastp^65^ and the results were retrieved using custom Python scripts. All contigs were aligned to BLAST NR database using MEGABLAST^65^ and the consensus FASTA was generated by reordering contigs using MAUVE^38^. Annotation of a predicted ORF is based on the first best hit from the Blastp. The annotated genomes were uploaded to NCBI using BankIt web-based submission tool. The NCBI accession numbers are: KU877344.1 (PLMV), KU761889.1 (MVB), and KY073338 (KV). Accession numbers of the scaffolds from the draft assembly of BAV genome are available under the bioproject PRJNA429331. For reconstructing the phylogenies, amino acid sequences were aligned using ClustalO^66^ and trees were generated using FastTree^67^ with default parameters.

## Supplementary information

Supplementary info
