# Supplementary material for: Genomic and metagenomic signatures of giant viruses are ubiquitous in water samples from sewage, inland lake, waste water treatment plant, and municipal water supply in Mumbai, India
Source: Sci Rep. 2019 Mar 6;9:3690. doi: 10.1038/s41598-019-40171-y (PMC6403294; doi:10.1038/s41598-019-40171-y)
Supplement: Supplementary file 1 — Supplementary info [file 41598_2019_40171_MOESM1_ESM.pdf]

Genomic and metagenomic signatures of giant viruses are ubiquitous in water samples from sewage, inland lake, waste water treatment plant, and municipal water supply in Mumbai, India

Anirvan Chatterjee[1], *Thomas Sicheritz-Pontén*[2,3] , *Rajesh Yadav*[1] *and Kiran Kondabagil*[1]*
[1]Department of Biosciences and Bioengineering, Indian Institute of Technology Bombay, Powai, Mumbai, India.
[2] Department of Bio and Health Informatics, Technical University of Denmark, Lyngby, Denmark.
[3]Centre of Excellence for Omics-Driven Computational Biodiscovery (COMBio), Faculty of Applied Sciences, AIMST University, Kedah, Malaysia

Key words: giant virus; metagenomics; WWTP; municipal water supply

Running Title: giant viruses in Mumbai

*Corresponding author
Kiran Kondabagil
Department of Biosciences and Bioengineering
Indian Institute of Technology Bombay, Powai
Mumbai, Maharashtra 400076, India.

Fax: +91(22) 25723480
Email: kirankondabagil@iitb.ac.in ; kirankondabagil@gmail.com

Supplementary Table 1: List of viruses with genome length > 300,000 bp used to generated blast databse for querying the metagenomes

NCBI Accession Number	Taxonomic ID	Genome Length (in bps)
AFYC0100000	Acanthamoeba polyphaga lentillevirus isolate lvs contig00005	788571
JF801956.1	Acanthamoeba castellanii mamavirus strain Hal-V	1191693
AP017645.1	Acanthamoeba castellanii mimivirus DNA	1182849
AP017644.1	Acanthamoeba castellanii mimivirus DNA	1182801
NC_014649.1	Acanthamoeba polyphaga mimivirus	1181549
HQ336222.2	Acanthamoeba polyphaga mimivirus	1181549
AY653733.1	Acanthamoeba polyphaga mimivirus	1181404
JN036606.1	Acanthamoeba polyphaga mimivirus isolate M4	981813
MF405918.1	Acanthamoeba polyphaga mimivirus isolate Tupan deep ocean	1516267
KY523104.1	Acanthamoeba polyphaga mimivirus isolate Tupanvirus	1439508
JX962719.1	Acanthamoeba polyphaga moumouvirus	1021348
NC_020104.1	Acanthamoeba polyphaga moumouvirus	1021348
JX997156.1	Acanthocystis turfacea Chlorella virus Can0610SP	306752
JX997168.1	Acanthocystis turfacea Chlorella virus GM0701.1	315239
JX997174.1	Acanthocystis turfacea Chlorella virus MN0810.1	327406
JX997177.1	Acanthocystis turfacea Chlorella virus NE-JV-2	319583
JX997180.1	Acanthocystis turfacea Chlorella virus NTS-1	323517
JX997185.1	Acanthocystis turfacea Chlorella virus OR0704.3	311647
JX997186.1	Acanthocystis turfacea Chlorella virus TN603.4.2	320880
MF403008.1	Agrobacterium phage Atu_ph07	490380
KR819915.1	Apis mellifera filamentous virus isolate CH-CO5	496396
NC_027925.1	Apis mellifera filamentous virus isolate CH-CO5	496396
KJ645900.1	Aureococcus anophagefferens virus isolate BtV-01	370920
NC_024697.1	Aureococcus anophagefferens virus isolate BtV-01	370920
JN638751.1	Bacillus phage G	497513
NC_023719.1	Bacillus phage G	497513
MF782455.1	Bodo saltans virus strain NG1	1385869
NC_029692.1	Brazilian marseillevirus strain BH2014	362276
KT752522.1	Brazilian marseillevirus strain BH2014	362276
NC_014637.1	Cafeteria roenbergensis virus BV-PW1	617453
GU244497.1	Cafeteria roenbergensis virus BV-PW1	617453
NC_005309.1	Canarypox virus	359853
AY318871.1	Canarypox virus strain ATCC VR-111	359853
KF261120.1	Cannes 8 virus	374041
KY684083.1	Catovirus CTV1 Catovirus_1 genomic sequence	1152313
KY684084.1	Catovirus CTV1 Catovirus_2 genomic sequence	379946
NC_032108.1	Cedratvirus A11 genome assembly	589068
LT671577.1	Cedratvirus A11 genome assembly	589068
LT907979.1	Cedratvirus lausannensis genome assembly	575161
HF679132.1	Choristoneura biennis entomopoxvirus 'L'	307691

NC_021248.1	Choristoneura biennis entomopoxvirus 'L'	307691
KT820662.1	Chrysochromulina ericina virus isolate CeV-01B	473558
NC_028094.1	Chrysochromulina ericina virus isolate CeV-01B	473558
HF586473.1	Cotesia congregata sequence containing Cotesia congregata bracovirus proviral locus 2 (PL2)	522749
JN882285.1	Cronobacter phage vB_CsaM_GAP32	358663
NC_019401.1	Cronobacter phage vB_CsaM_GAP32	358663
AF204951.2	Ectocarpus siliculosus virus	335593
NC_002687.1	Ectocarpus siliculosus virus 1	335593
KF481686.1	Emiliania huxleyi virus 145 partial genome sequence	399873
KF481687.1	Emiliania huxleyi virus 156 partial genome sequence	400341
KF481688.1	Emiliania huxleyi virus 164 partial genome sequence	401558
KF481685.1	Emiliania huxleyi virus 18 partial genome sequence	400762
JF974311.1	Emiliania huxleyi virus 201	407301
HQ634145.1	Emiliania huxleyi virus 202	407516
JF974291.1	Emiliania huxleyi virus 203	400520
JF974317.1	Emiliania huxleyi virus 207	421891
JF974318.1	Emiliania huxleyi virus 208	411003
JF974290.1	Emiliania huxleyi virus 84	396620
NC_007346.1	Emiliania huxleyi virus 86	407339
AJ890364.1	Emiliania huxleyi virus 86 isolate EhV86	407339
JF974310.1	Emiliania huxleyi virus 88	397298
FN429076.1	Emiliania huxleyi virus 99B1 draft genome sequence	376759
HQ634146.1	Emiliania huxleyi virus PS401	301520
NC_019526.1	Enterobacteria phage vB_KleM-RaK2	345809
JQ513383.1	Enterobacteria phage vB_KleM-RaK2	345809
NC_025447.1	Escherichia phage 121Q	348532
KM507819.1	Escherichia phage 121Q	348532
NC_027364.1	Escherichia phage PBECO 4	348113
KC295538.1	Escherichia phage PBECO 4	348113
LT603033.1	Escherichia phage vB_Eco_slurp01 genome assembly	348043
LT839607.1	Faustovirus ST1 genome assembly	470659
KU556803.1	Faustovirus strain D3	455803
KU702950.1	Faustovirus strain D5a	466051
KU702949.1	Faustovirus strain D5b	464523
KU702951.1	Faustovirus strain D6	462011
KJ614390.1	Faustovirus strain E12	466265
KU702952.1	Faustovirus strain E23	465956
KU702948.1	Faustovirus strain E24	466012
KT835053.1	Golden Marseillevirus	360610
NC_031465.1	Golden Marseillevirus	360610
KF493731.1	Hirudovirus strain Sangsue	1181042
KY684103.1	Hokovirus HKV1 Hokovirus_1 genomic sequence	450695
KY684104.1	Hokovirus HKV1 Hokovirus_2 genomic sequence	317278

KY684105.1	Hokovirus HKV1 Hokovirus_3 genomic sequence	407933
KF527888.1	Insectomime virus strain V478	386631
HG428764.1	Insectomime virus V478 genomic scaffold	382785
KX552040.1	Kaumoebavirus isolate Sc	350731
NC_034249.1	Kaumoebavirus isolate Sc	350731
AB897757.1	Klebsiella phage K64-1 DNA	346602
NC_027399.1	Klebsiella phage K64-1 DNA	346602
KY684108.1	Klosneuvirus KNV1 Klosneuvirus_1 genomic sequence	451653
KY684109.1	Klosneuvirus KNV1 Klosneuvirus_2 genomic sequence	333228
KY073338.1	Kurlavirus BKC-1	361368
NC_015326.1	Lausannevirus	346754
HQ113105.1	Lausannevirus isolate 7715	346754
NC_013756.1	Marseillevirus marseillevirus strain T19	368454
GU071086.1	Marseillevirus marseillevirus strain T19	368454
LN867403.1	Megavirus avenue9 genome assembly Megavirus avenue9	1214278
LN868256.1	Megavirus battle43 genome assembly Megavirus battle43	1226044
LN868539.1	Megavirus bus genome assembly Megavirus bus	1229128
NC_016072.1	Megavirus chiliensis	1259197
JN258408.1	Megavirus chiliensis	1259197
JX975216.1	Megavirus courdo11	1246126
LN868540.1	Megavirus courdo5 genome assembly Megavirus courdo5	921986
JN885991.1	Megavirus courdo7 isolate Mv13-c7	529672
JX885207.1	Megavirus lba isolate LBA111	1230522
LN868518.1	Megavirus montpellier3 genome assembly Megavirus montpellier3	1242698
LN868520.1	Megavirus shan genome assembly Megavirus shan scaffold scaffold00002	1258603
LN868526.1	Megavirus T1 genome assembly Megavirus T1	1216769
LN869537.1	Megavirus T4 genome assembly Megavirus T4	970404
KF527229.1	Megavirus terra1 genome	1244621
KM275475.1	Melbournevirus isolate 1	369360
NC_025412.1	Melbournevirus isolate 1	369360
LN867402.1	Mimivirus amazonia genome assembly Mimivirus amazonia	1179120
KU761889.1	Mimivirus Bombay isolate 1 genomic sequence	1182200
LN871163.1	Mimivirus fauteuil genome assembly Mimivirus fauteuil	1180980
LN871173.1	Mimivirus longchamps genome assembly Mimivirus longchamps	1104869
LN871174.1	Mimivirus pointerouge1 genome assembly Mimivirus pointerouge1	1150884
LN871172.1	Mimivirus pointerouge2 genome assembly Mimivirus pointerouge2	1162847
KF527228.1	Mimivirus terra2 genome	1168989
NC_023639.1	Mimivirus terra2 genome	1168989
KR921745.1	Mollivirus sibericum isolate P1084-T	651523
NC_027867.1	Mollivirus sibericum isolate P1084-T	651523
LN871171.1	Moumouvirus battle49 genome assembly Moumouvirus battle49	844927

JN885998.1	Moumouvirus Monve isolate Mv13-mv	345413
KT599914.1	Niemeyer virus	1299140
KX066233.1	Noumeavirus isolate NMV1	376207
NC_033775.1	Noumeavirus isolate NMV1	376207
KX857749.1	Only Syngen Nebraska virus 5	327147
NC_032001.1	Only Syngen Nebraska virus 5	327147
HQ704802.1	Organic Lake phycodnavirus 1 genomic sequence	344723
NC_036594.1	Orpheovirus IHUMI-LCC2 genome assembly	1473573
LT906555.1	Orpheovirus IHUMI-LCC2 genome assembly	1473573
LT706986.1	Pacmanvirus A23 genome assembly	395405
NC_034383.1	Pacmanvirus A23 genome assembly	395405
KC977570.1	Pandoravirus dulcis	1908524
NC_021858.1	Pandoravirus dulcis	1908524
KP136319.1	Pandoravirus inopinatum isolate KlaHel	2243109
NC_026440.1	Pandoravirus inopinatum isolate KlaHel	2243109
NC_022098.1	Pandoravirus salinus	2473870
KC977571.1	Pandoravirus salinus	2473870
NC_000852.5	Paramecium bursaria Chlorella virus 1	330611
JF411744.1	Paramecium bursaria Chlorella virus 1 (PBCV-1)	330611
JX997153.1	Paramecium bursaria Chlorella virus AN69C	332309
JX997154.1	Paramecium bursaria Chlorella virus AP110A	327349
NC_009899.1	Paramecium bursaria Chlorella virus AR158	344691
DQ491003.2	Paramecium bursaria Chlorella virus AR158 genomic sequence	344691
JX997157.1	Paramecium bursaria Chlorella virus Can18-4	329890
JX997159.1	Paramecium bursaria Chlorella virus CVA-1	326592
JX997160.1	Paramecium bursaria Chlorella virus CVB-1	319457
JX997161.1	Paramecium bursaria Chlorella virus CVG-1	318742
JX997162.1	Paramecium bursaria Chlorella virus CviKI	309195
JX997163.1	Paramecium bursaria Chlorella virus CVM-1	327107
JX997164.1	Paramecium bursaria Chlorella virus CVR-1	330002
JX997165.1	Paramecium bursaria Chlorella virus CvsA1	311223
JX997166.1	Paramecium bursaria Chlorella virus CZ-2	305715
NC_008603.1	Paramecium bursaria Chlorella virus FR483	321240
DQ890022.1	Paramecium bursaria Chlorella virus FR483	321240
JX997167.1	Paramecium bursaria Chlorella virus Fr5L	303810
JX997169.1	Paramecium bursaria Chlorella virus IL-3A	323497
JX997170.1	Paramecium bursaria Chlorella virus IL-5-2s1	345255
JX997172.1	Paramecium bursaria Chlorella virus MA-1D	339653
JX997173.1	Paramecium bursaria Chlorella virus MA-1E	339391
DQ491001.1	Paramecium bursaria chlorella virus MT325 genomic sequence	314335
JX997176.1	Paramecium bursaria Chlorella virus NE-JV-1	326559
JX997179.1	Paramecium bursaria Chlorella virus NE-JV-4	328315
JX997181.1	Paramecium bursaria Chlorella virus NW665.2	325996
NC_009898.1	Paramecium bursaria Chlorella virus NY2A	368683

DQ491002.1	Paramecium bursaria Chlorella virus NY2A Chlorella virus NY2A ctg_13 genomic sequence	368683
JX997182.1	Paramecium bursaria Chlorella virus NY-2B	344863
JX997183.1	Paramecium bursaria Chlorella virus NYs1	348463
JX997184.1	Paramecium bursaria Chlorella virus OR0704.2.2	313584
KU574722.1	Pectobacterium phage CBB	378379
KJ859677.1	Penguinpox virus isolate PSan92	306862
NC_024446.1	Penguinpox virus isolate PSan92	306862
HQ634147.1	Phaeocystis globosa virus 12T	460002
HQ634144.1	Phaeocystis globosa virus 14T	452892
NC_021312.1	Phaeocystis globosa virus strain 16T	459984
KC662249.1	Phaeocystis globosa virus strain 16T	459984
NC_023423.1	Pithovirus sibericum isolate P1084-T	610033
KF740664.1	Pithovirus sibericum isolate P1084-T	610033
LT598836.1	Pithovirus sp. LC8 genome assembly	683254
KT428292.1	Port-miou virus	349275
KU877344.1	Powai lake megavirus isolate 1	1208707
EU197055.1	Pseudomonas phage 201phi2-1	316674
NC_010821.1	Pseudomonas phage 201phi2-1	316674
MF042360.1	Pseudomonas phage Phabio	309157
NC_028999.1	Pseudomonas phage PhiPA3	309208
HQ630627.1	Pseudomonas phage PhiPA3	309208
MF360958.1	Salicola phage SCTP-2	440001
KF959826.2	Samba virus	1181380
KY110734.1	Saudi moumouvirus	1030056
AX151396.1	Sequence 1 from Patent WO0138351	305107
KY630187.1	Serratia phage BF	357154
KX857216.1	Shearwaterpox virus strain SWPV-1	326929
KX857215.1	Shearwaterpox virus strain SWPV-2	351108
AF440570.1	Shrimp white spot syndrome virus	307287
NC_030230.1	Tokyovirus A1 DNA	372707
AP017398.1	Tokyovirus A1 DNA	372707
KF483846.1	Tunisvirus fontaine2 strain U484	380011
KM982403.1	UNVERIFIED: Acanthamoeba polyphaga mimivirus strain Amazonia	1179119
KM982402.1	UNVERIFIED: Acanthamoeba polyphaga mimivirus strain Kroon	1221932
KM982401.1	UNVERIFIED: Acanthamoeba polyphaga mimivirus strain Oyster	1200220
KC008572.1	UNVERIFIED: Moumouvirus goulette	1016844
MG592671.1	Vibrio phage 2.275.O._10N.286.54.E11	348911
AF332093.3	White spot syndrome virus	305119
KR083866.1	White spot syndrome virus isolate EG3	305119
KT995472.1	White spot syndrome virus strain CN01	309286
NC_003225.3	White spot syndrome virus strain CN01	309286
AP018399.1	Xanthomonas phage XacN1 DNA	384670
LT960552.1	Yersinia phage fHe-Yen9-03 genome assembly	352596

LT960551.1	Yersinia phage fHe-Yen9-04 genome assembly	354378

Supplementary Table 2: Top hits of giant viruses in pre-filter

NCBI Accession Number	Taxonomic name	Blast Score	E-Value
HF679132.1	Choristoneura biennis entomopoxvirus 'L'	55.4	0.0006
JQ360576.1	Chimpanzee alpha-1 herpesvirus strain 105640	57.2	0.0002
KU893109.1	Human herpesvirus 2 isolate 2012-9333, partial genome	57.2	0.0002
DQ517337.1	Trichoplusia ni ascovirus 2c, complete genome	59	0.00005
HG425349.2	Spodoptera exigua multiple nucleopolyhedrovirus	60.8	0.00001
AY386371.1	Yaba monkey tumor virus, complete genome	60.8	0.00001
KR921745.1	Mollivirus sibericum isolate P1084-T, complete genome	62.6	0.000004
DQ123841.1	Agrotis segetum nucleopolyhedrovirus, complete genome	68	1E-07
AF033815.1	Mason-Pfizer monkey virus, complete genome	66.2	2E-08
M12349.1	Simian Mason-Pfizer D-type retrovirus (MPMV/6A)	66.2	2E-08
AB107976.1	Chlorella virus k2-gfat gene for glutamine:fructose-6	71.6	8E-09
KP296186.1	Diatraea saccharalis granulovirus, complete genome	71.6	8E-09
KF158713.1	Hemileuca sp. nucleopolyhedrovirus, complete genome	71.6	8E-09
DQ453159.1	Geobacillus virus E2, complete genome	73.4	2E-09
EF524222.1	Shrimp white spot syndrome virus unknown gene	73.4	2E-09
AP013055.1	Anomala cuprea entomopoxvirus DNA, complete genome	75.2	7E-10
AF246223.1	Mycoplasma virus P1, complete genome	75.2	7E-10
KC662249.1	Phaeocystis globosa virus strain 16T, complete genome	77	1E-10
HF920633.1	Invertebrate iridovirus 22 complete genome	78.8	6E-11
JF411744.1	Paramecium bursaria Chlorella virus 1 (PBCV-1)	78.8	1E-11
HQ632826.1	Micromonas pusilla virus 12T genomic sequence	82.4	5E-12
KT835053.1	Golden Marseillevirus, complete genome	82.4	4E-12
KR083866.1	White spot syndrome virus isolate EG3, complete genome	82.4	4E-12
AY009089.1	Camelpox virus CMS, complete genome	84.2	1E-12
HF920634.1	Invertebrate iridescent virus 22 isolate IIV22	84.2	1E-12
L22579.1	Variola major virus (strain Bangladesh-1975)	84.2	1E-12
KR819915.1	Apis mellifera filamentous virus isolate CH-CO5	86	4E-13
AY225133.1	Feldmannia irregularis virus a strain FirrV-1 contig	86	4E-13
KM009991.1	Peridroma alphabaculovirus isolate GR_167	86	2E-13
KC540776.1	Uncultured virus isolate P4 ribonucleotide reductase	86	2E-13
KU199244.1	Crucian carp herpesvirus isolate 1301, complete genome	87.8	1E-13
AY689437.1	Deerpox virus W-1170-84, complete genome	87.8	1E-13
DQ437594.1	Taterapox virus strain Dahomey 1968, complete genome	87.8	1E-13
GU244497.1	Cafeteria roenbergensis virus BV-PW1, complete genome	89.7	3E-14
AY318871.1	Canarypox virus strain ATCC VR-111, complete genome	89.7	3E-14
EU522111.1	Musca domestica salivary gland hypertrophy virus	89.7	2E-14
KX857749.1	Only Syngen Nebraska virus 5, complete genome	89.7	2E-14
KP874736.1	Ostreococcus lucimarinus virus 2 isolate Olv2	89.7	2E-14
AJ920288.1	Euproctis pseudoconspersa nucleopolyhedrovirus p24	91.5	9E-15

HM004429.1	Micromonas sp. RCC1109 virus MpV1, complete genome	93.3	3E-15
AF170722.1	Rabbit fibroma virus, complete genome	93.3	2E-15
AY484669.1	Rabbitpox virus, complete genome	96.9	2E-16
AF095689.1	Vaccinia virus (strain Tian Tan) complete genome	96.9	2E-16
EF101928.1	Acanthocystis turfacea Chlorella virus 1	96.9	1E-16
LC015647.1	Yellowstone lake phycodnavirus 1 DNA	98.7	6E-17
JQ410350.1	Ectromelia virus ERPV culture-collection ATCC:VR-1431	102	5E-18
DQ792504.1	Horsepox virus isolate MNR-76, complete genome	102	5E-18
LN864566.1	Cowpox virus complete genome, strain FM2292	105	4E-19
KP849469.1	Monkeypox virus isolate Boende_DRC_2008, complete genome	105	4E-19
KU877344.1	Powai lake megavirus isolate 1, complete genome	107	1E-19
AP006270.1	Adoxophyes honmai nucleopolyhedrovirus DNA, complete	109	3E-20
HM004432.1	Bathycoccus sp. RCC1105 virus BpV1, complete genome	109	3E-20
KX027736.1	Anguillid herpesvirus 1 isolate HVA980811	114	8E-22
KU556803.1	Faustovirus strain D3, complete genome	114	8E-22
KJ645900.1	Aureococcus anophagefferens virus isolate BtV-01	116	2E-22
HF679134.1	Mythimna separata entomopoxvirus 'L', complete genome	116	2E-22
KY094066.1	BeAn 58058 virus, complete genome	118	6E-23
LT671577.1	Cedratvirus A11 genome assembly	118	6E-23
KF481686.1	Emiliania huxleyi virus 145 partial genome sequence	118	6E-23
JX975216.1	Megavirus courdo11, complete genome	118	3E-23
JN258408.1	Megavirus chiliensis, complete genome	122	2E-24
JN885991.1	Megavirus courdo7 isolate Mv13-c7, partial genome	122	2E-24
JX885207.1	Megavirus lba isolate LBA111, complete genome	122	2E-24
KF527229.1	Megavirus terra1 genome	122	2E-24
AY208990.1	Carp nephritis and gill necrosis virus genomic fragment	123	1E-24
EU001284.1	Glyptapanteles flavicoxis bracovirus segment 29	122	1E-24
JQ815363.1	Cyprinid herpesvirus 1 strain NG-J1, complete genome	125	2E-25
AY786308.2	Koi herpesvirus ribonucleotide reductase gene	127	1E-25
JX962719.1	Acanthamoeba polyphaga moumouvirus, complete genome	127	6E-26
JN885998.1	Moumouvirus Monve isolate Mv13-mv, partial genome	127	6E-26
HQ113105.1	Lausannevirus isolate 7715, complete genome	132	3E-27
KT428292.1	Port-miou virus, complete genome	132	3E-27
KX618634.1	Lymantria dispar multiple nucleopolyhedrovirus	132	2E-27
AP017398.1	Tokyovirus A1 DNA, nearly complete genome	140	7E-30
AP017645.1	Acanthamoeba castellanii mimivirus DNA	141	6E-30
KU761889.1	Mimivirus Bombay isolate 1 genomic sequence	141	6E-30
KT599914.1	Niemeyer virus, partial genome	141	6E-30
KF959826.2	Samba virus, complete genome	141	6E-30
EU552531.1	Recombinant virus 6918VP60-T2, complete sequence	141	5E-30
KC951854.1	Goatpox virus FZ, complete genome	143	2E-30
JX565584.1	Myxoma virus isolate BD23, complete genome	143	2E-30
KU749310.1	Skunkpox virus strain WA, complete genome	143	2E-30
KJ859677.1	Penguinpox virus isolate PSan92, complete genome	145	2E-31
JF801956.1	Acanthamoeba castellanii mamavirus strain Hal-V	147	1E-31
KF493731.1	Hirudovirus strain Sangsue, complete genome	147	1E-31

KF527228.1	Mimivirus terra2 genome	147	1E-31
KC977570.1	Pandoravirus dulcis, complete genome	147	1E-31
KP136319.1	Pandoravirus inopinatum isolate KlaHel, complete genome	149	4E-32
KT820662.1	Chrysochromulina ericina virus isolate CeV-01B	152	3E-33
KU749309.1	Raccoonpox virus strain 85A, complete genome	152	3E-33
AF063866.1	Melanoplus sanguinipes entomopoxvirus, complete genome	154	9E-34
HQ704802.1	Organic Lake phycodnavirus 1 genomic sequence	168	4E-38
KC977571.1	Pandoravirus salinus, complete genome	168	4E-38
AF065756.1	Stealth virus 1 clone 3B43 T7	291	5E-75

Supplementary Table 3: Top hits of giant viruses in WWTP Dry Bed

NCBI Accession Number	Taxonomic name	Blast Score	E-Value
HM004432.1	Bathycoccus sp. RCC1105 virus BpV1, complete genome	57.2	2.00E-04
AF198100.1	Fowlpox virus, complete genome	57.2	2.00E-04
KT159937.1	Salmon gill poxvirus, complete genome	57.2	2.00E-04
AF410153.1	Swinepox virus isolate 17077-99, complete genome	59	5.00E-05
KR921745.1	Mollivirus sibericum isolate P1084-T, complete genome	60.8	1.00E-05
AY386371.1	Yaba monkey tumor virus, complete genome	62.6	4.00E-06
AP009046.1	Hyphantria cunea nucleopolyhedrovirus genomic DNA	64.4	1.00E-06
JX997183.1	Paramecium bursaria Chlorella virus NYs1, partial genome	64.4	1.00E-06
FR719956.1	Roseovarius Plymouth Podovirus 1 partial sequence	64.4	1.00E-06
JX997187.1	Acanthocystis turfacea Chlorella virus WI0606	66.2	3.00E-07
KJ859677.1	Penguinpox virus isolate PSan92, complete genome	66.2	3.00E-07
KJ801920.1	Pigeonpox virus isolate FeP2, complete genome	66.2	3.00E-07
AF170722.1	Rabbit fibroma virus, complete genome	69.8	3.00E-08
DQ643392.1	Aedes taeniorhynchus iridescent virus, complete genome	71.6	8.00E-09
KX857749.1	Only Syngen Nebraska virus 5, complete genome	71.6	8.00E-09
GU244497.1	Cafeteria roenbergensis virus BV-PW1, complete genome	71.6	6.00E-09
AY208990.1	Carp nephritis and gill necrosis virus genomic fragment	73.4	2.00E-09
KP747440.1	Dasychira pudibunda nucleopolyhedrovirus isolate ML1	73.4	2.00E-09
AF325155.1	Spodoptera litura nucleopolyhedrovirus strain G2	73.4	2.00E-09
AF012825.2	Ectromelia virus strain Moscow, complete genome	75.2	7.00E-10
AF380138.1	Monkeypox virus strain Zaire-96-I-16, complete genome	75.2	7.00E-10
KC662249.1	Phaeocystis globosa virus strain 16T, complete genome	77	2.00E-10
AF440570.1	Shrimp white spot syndrome virus, complete genome	77	2.00E-10
AF332093.3	White spot syndrome virus, complete genome	77	2.00E-10
HQ420900.1	Cowpox virus strain UK2000_K2984, complete genome	80.6	2.00E-11
DQ792504.1	Horsepox virus isolate MNR-76, complete genome	80.6	2.00E-11
AY484669.1	Rabbitpox virus, complete genome	80.6	2.00E-11
KF179385.1	Vaccinia virus, complete genome	80.6	2.00E-11
HQ632826.1	Micromonas pusilla virus 12T genomic sequence	82.4	4.00E-12
DQ657948.1	Cyprinid herpesvirus 3 strain KHV-U, complete genome	82.4	3.00E-12
KT835053.1	Golden Marseillevirus, complete genome	84.2	1.00E-12
JF801956.1	Acanthamoeba castellanii mamavirus strain Hal-V	86	4.00E-13
KU702951.1	Faustovirus strain D6, complete genome	87.8	1.00E-13
KP768318.1	Camelpox virus strain 0408151v, complete genome	89.7	3.00E-14
HQ336222.2	Acanthamoeba polyphaga mimivirus, complete genome	91.5	9.00E-15
KT820662.1	Chrysochromulina ericina virus isolate CeV-01B	91.5	9.00E-15
KF493731.1	Hirudovirus strain Sangsue, complete genome	91.5	9.00E-15
KU761889.1	Mimivirus Bombay isolate 1 genomic sequence	91.5	9.00E-15
KF527228.1	Mimivirus terra2 genome	91.5	9.00E-15
KT599914.1	Niemeyer virus, partial genome	91.5	9.00E-15
KF959826.2	Samba virus, complete genome	91.5	9.00E-15
DQ437594.1	Taterapox virus strain Dahomey 1968, complete genome	93.3	3.00E-15

DQ441448.1	Variola virus strain Yugoslavia 1972 V72-164	93.3	3.00E-15
JX975216.1	Megavirus courdo11, complete genome	100	2.00E-17
JN885991.1	Megavirus courdo7 isolate Mv13-c7, partial genome	100	2.00E-17
KF527229.1	Megavirus terra1 genome	100	2.00E-17
KU877344.1	Powai lake megavirus isolate 1, complete genome	105	4.00E-19
AJ890364.1	Emiliania huxleyi virus 86 isolate EhV86	109	3.00E-20
KT752522.1	Brazilian marseillevirus strain BH2014, complete genome	113	3.00E-21
KJ645900.1	Aureococcus anophagefferens virus isolate BtV-01	118	6.00E-23
JX885207.1	Megavirus lba isolate LBA111, complete genome	118	6.00E-23
KP136319.1	Pandoravirus inopinatum isolate KlaHel, complete genome	122	5.00E-24
JN258408.1	Megavirus chiliensis, complete genome	127	1.00E-25
KR029585.1	Uncultured marine virus isolate Anoxic2_1	127	1.00E-25
KC977570.1	Pandoravirus dulcis, complete genome	141	6.00E-30
KC977571.1	Pandoravirus salinus, complete genome	161	6.00E-36
JX962719.1	Acanthamoeba polyphaga moumouvirus	187	1.00E-43
JN885999.1	Moumouvirus Monve isolate Mv13-mv, partial genome	187	1.00E-43
AF191073.1	Stealth virus 1 clone 3B43, genomic sequence	291	5.00E-75

Supplementary Table 4: Top hits of giant viruses in WWTP sludge

NCBI Accession Number	Taxonomic name	Blast Score	E-Value
X72087.1	Aujeszky's disease virus RR1 and RR2 genes	57.2	2.00E-04
KT832477.1	Elephant endotheliotropic herpesvirus 4 isolate	57.2	2.00E-04
KU552118.1	Suid alphaherpesvirus 1 isolate LA, complete genome	57.2	2.00E-04
KU360259.1	Suid herpesvirus 1 isolate DL14/08, complete genome	57.2	2.00E-04
BK001744.1	TPA: Suid herpesvirus 1, complete genome	57.2	2.00E-04
KT804738.1	Giant seaperch iridovirus isolate GSIV-K1	57.2	1.00E-04
AY894343.1	Orange-spotted grouper iridovirus, complete genome	57.2	1.00E-04
KC244182.1	Rock bream iridovirus isolate RBIV-C1, complete genome	57.2	1.00E-04
LC015648.1	Yellowstone lake phycodnavirus 2 DNA	59	5.00E-05
KM609482.1	Autographa californica multiple nucleopolyhedrovirus	59	4.00E-05
KU697903.1	Mutant Autographa californica nucleopolyhedrovirus	59	4.00E-05
KP747440.1	Dasychira pudibunda nucleopolyhedrovirus isolate ML1	60.8	1.00E-05
AP009046.1	Hyphantria cunea nucleopolyhedrovirus genomic DNA	60.8	1.00E-05
KF527888.1	Insectomime virus strain V478, partial genome	60.8	1.00E-05
KP752043.1	Lambdina fiscellaria nucleopolyhedrovirus isolate GR1	60.8	1.00E-05
KT428292.1	Port-miou virus, complete genome	60.8	1.00E-05
AY145471.1	Rachiplusia ou multiple nucleopolyhedrovirus	60.8	1.00E-05
KF483846.1	Tunisvirus fontaine2 strain U484, complete genome	60.8	1.00E-05
X98924.1	Spodoptera littoralis nuclear polyhedrosis virus gene	62.6	2.00E-06
D14469.1	Chlorella virus CVK1F1 gene, repeat region	64.4	1.00E-06
DQ123841.1	Agrotis segetum nucleopolyhedrovirus, complete genome	64.4	5.00E-07
KJ003983.1	IAS virus, complete genome	66.2	3.00E-07
KC117377.1	Halovirus HVTV-1, complete genome	68	1.00E-07
KR921745.1	Mollivirus sibericum isolate P1084-T, complete genome	68	1.00E-07
AF204951.2	Ectocarpus siliculosus virus, complete genome	69.8	2.00E-08
GU244497.1	Cafeteria roenbergensis virus BV-PW1, complete genome	73.4	2.00E-09
AY689437.1	Deerpox virus W-1170-84, complete genome	73.4	2.00E-09
KP188543.1	Dengue virus 1 isolate DENV1 BR/SJRP/484/2012	73.4	2.00E-09
AB018418.1	Red sea bream iridovirus genes for largest subunit	75.2	6.00E-10
HF679132.1	Choristoneura biennis entomopoxvirus 'L'	77	2.00E-10
EU001267.1	Glyptapanteles flavicoxis bracovirus segment 10	77	2.00E-10
KU877344.1	Powai lake megavirus isolate 1, complete genome	75.2	2.00E-10
DQ517337.1	Trichoplusia ni ascovirus 2c, complete genome	77	2.00E-10
KT781098.1	Infectious spleen and kidney necrosis virus strain RS	78.8	5.00E-11
KY215944.1	Bovine alphaherpesvirus 1 isolate 216 II	80.6	2.00E-11
EF101928.1	Acanthocystis turfacea Chlorella virus 1	82.4	5.00E-12
KF261120.1	Cannes 8 virus, complete genome	84.2	1.00E-12
GU071086.1	Marseillevirus marseillevirus strain T19	84.2	1.00E-12
KM275475.1	Melbournevirus isolate 1, complete genome	84.2	1.00E-12
JF909601.1	Senegalvirus SSV-A contig6 genomic sequence	84.2	1.00E-12
KX853510.1	Uncultured virus isolate Rctr197k, partial genome	84.2	1.00E-12
EU522111.1	Musca domestica salivary gland hypertrophy virus	87.8	1.00E-13

JF411744.1	Paramecium bursaria Chlorella virus 1 (PBCV-1)	87.8	1.00E-13
GQ273492.1	Turbot reddish body iridovirus, complete genome	87.8	1.00E-13
KF481686.1	Emiliania huxleyi virus 145 partial genome sequence	87.8	8.00E-14
KR819915.1	Apis mellifera filamentous virus isolate CH-CO5	89.7	3.00E-14
KX857749.1	Only Syngen Nebraska virus 5, complete genome	89.7	3.00E-14
JX962719.1	Acanthamoeba polyphaga moumouvirus, complete genome	91.5	9.00E-15
KX618634.1	Lymantria dispar multiple nucleopolyhedrovirus	93.3	3.00E-15
JN885999.1	Moumouvirus Monve isolate Mv13-mv, partial genome	95.1	7.00E-16
EF420156.1	Tanapox virus isolate TPV-Kenya, complete genome	95.1	5.00E-16
AJ293568.1	Yaba-like disease virus (YLDV), complete genome	95.1	5.00E-16
AY208989.1	Carp nephritis and gill necrosis virus genomic fragment	96.9	2.00E-16
KT820662.1	Chrysochromulina ericina virus isolate CeV-01B	96.9	2.00E-16
AY786308.2	Koi herpesvirus ribonucleotide reductase gene	96.9	2.00E-16
LT671577.1	Cedratvirus A11 genome assembly, complete genome	100	2.00E-17
AP008984.1	Cyprinid herpesvirus 3 DNA, complete genome	102	5.00E-18
KJ645900.1	Aureococcus anophagefferens virus isolate BtV-01	109	3.00E-20
AF410153.1	Swinepox virus isolate 17077-99, complete genome	109	3.00E-20
KR029585.1	Uncultured marine virus isolate Anoxic2_1	113	3.00E-21
KU749309.1	Raccoonpox virus strain 85A, complete genome	114	7.00E-22
HQ849551.1	Yoka poxvirus strain DakArB 4268, complete genome	122	5.00E-24
AP017645.1	Acanthamoeba castellanii mimivirus DNA	123	1.00E-24
HQ336222.2	Acanthamoeba polyphaga mimivirus, complete genome	123	1.00E-24
KF493731.1	Hirudovirus strain Sangsue, complete genome	123	1.00E-24
KU761889.1	Mimivirus Bombay isolate 1 genomic sequence	123	1.00E-24
KF527228.1	Mimivirus terra2 genome	123	1.00E-24
KT599914.1	Niemeyer virus, partial genome	123	1.00E-24
KF959826.2	Samba virus, complete genome	123	1.00E-24
JF801956.1	Acanthamoeba castellanii mamavirus strain Hal-V	129	3.00E-26
JN258408.1	Megavirus chiliensis, complete genome	131	8.00E-27
JX975216.1	Megavirus courdo11, complete genome	131	8.00E-27
JN885991.1	Megavirus courdo7 isolate Mv13-c7, partial genome	131	8.00E-27
JX885207.1	Megavirus lba isolate LBA111, complete genome	131	8.00E-27
KF527229.1	Megavirus terra1 genome	131	8.00E-27
JQ410350.1	Ectromelia virus ERPV culture-collection ATCC:VR-1431	134	8.00E-28
KU749310.1	Skunkpox virus strain WA, complete genome	134	8.00E-28
KC977571.1	Pandoravirus salinus, complete genome	136	2.00E-28
KX027736.1	Anguillid herpesvirus 1 isolate HVA980811	138	7.00E-29
KU749311.1	Volepox virus strain CA, complete genome	138	7.00E-29
KP136319.1	Pandoravirus inopinatum isolate KlaHel, complete genome	161	6.00E-36
KC977570.1	Pandoravirus dulcis, complete genome	187	1.00E-43
AF065756.1	Stealth virus 1 clone 3B43 T7	417	#######

Supplementary Figure 1. Maximum likelihood based phylogeny of 4 Histone-like proteins (concatenated Fastas) in KUV. The phylogeny indicates a lineage specific sequence similarity, wherein KUV shares maximum homology with histone-like proteins in *Noumeavirus* belonging to *Marseilleviridae* Lineage B.

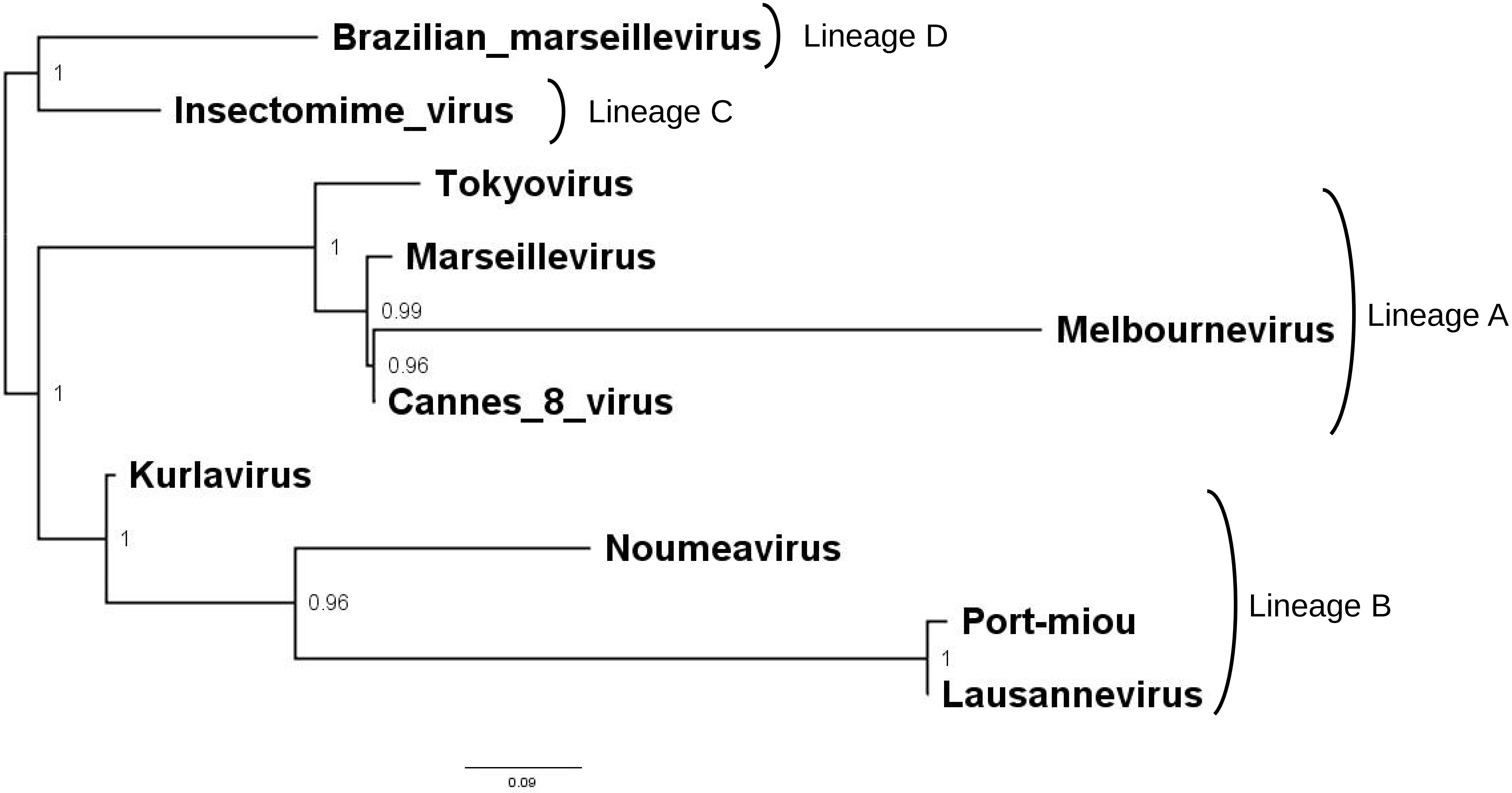

Supplementary Figure 2. Maximum unique matches (MUMs) matches among A) PLMV and other megaviruses, B) BAV and other megaviruses, C) MVB and other mimiviruses, and D) KUV and other marseilleviruses

MUMs were calculated using Nucmer, with a sliding window size of 100 nulceotides. Forward matches are shown in Red, reverse matches are shown in Blue.

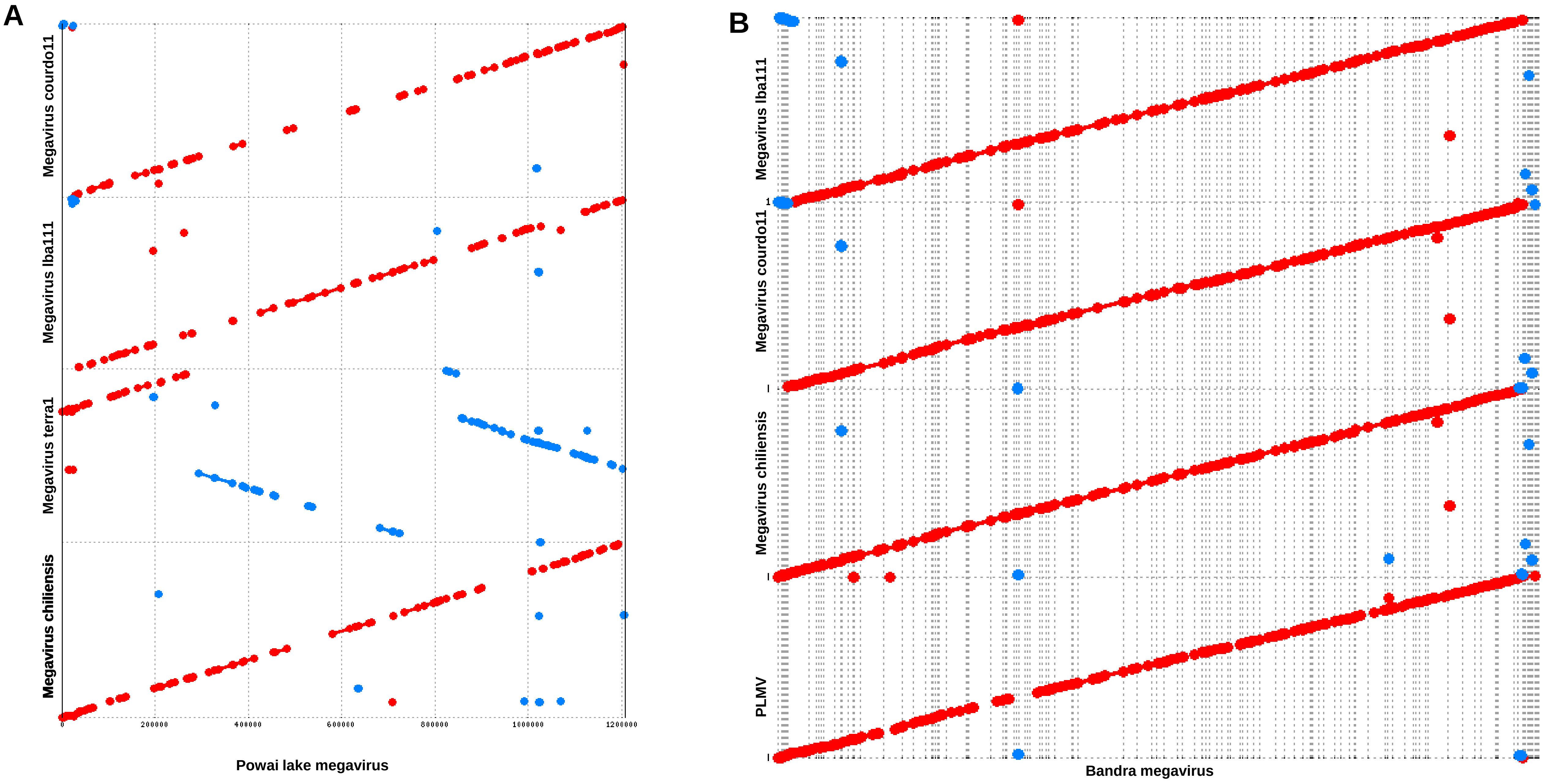

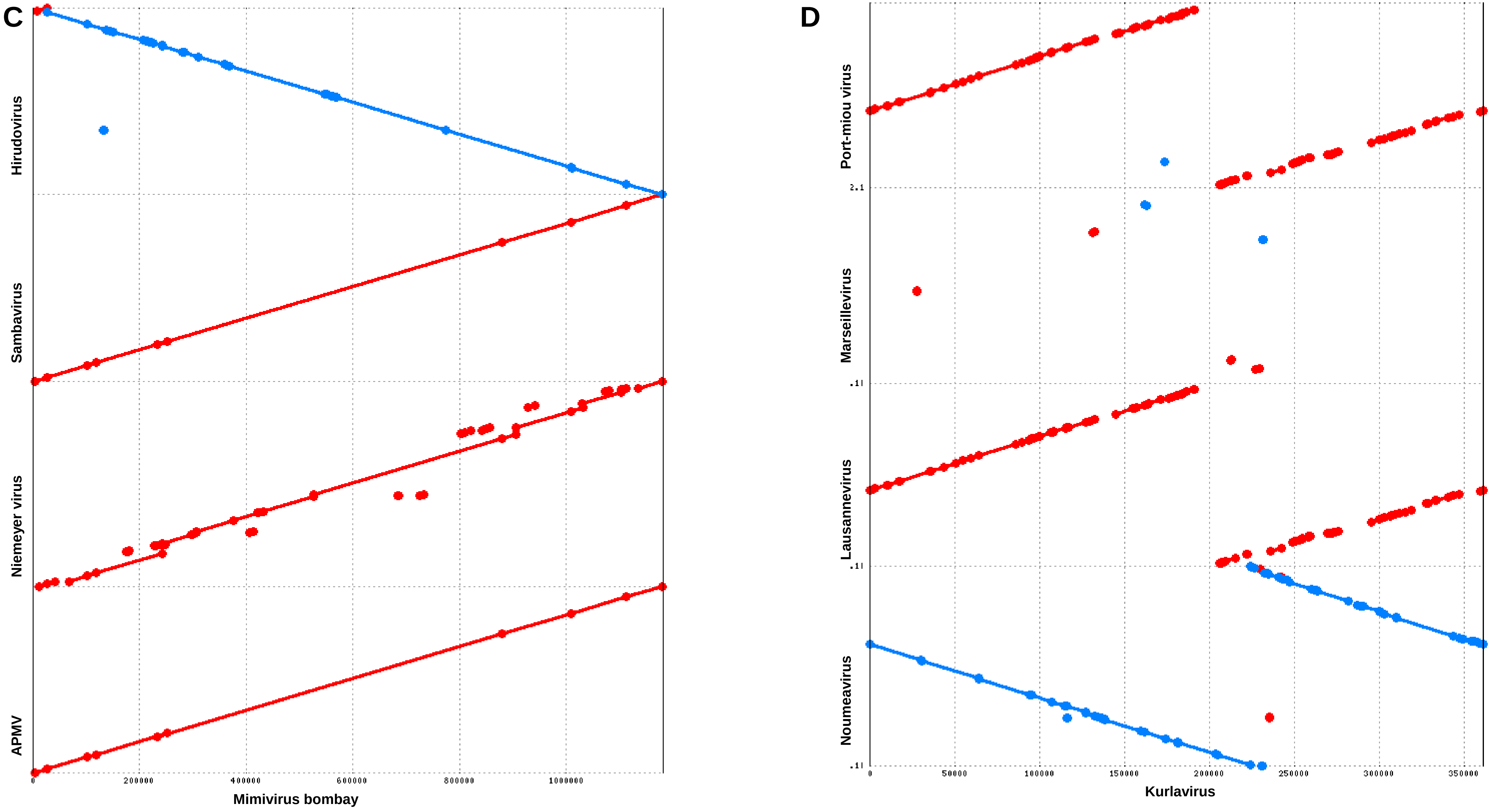

C
Hirudovirus
Sambavirus
Niemeyer virus
APMV
0
200000
400000
600000
800000
1000000
Mimivirus bombay
D
Port-miou virus
Marseillevirus
Lausannevirus
Noumeavirus
0
50000
100000
150000
200000
250000
300000
350000
Kurlavirus
